# Microhabitat Types Promote the Genetic Structure of a Micro-Endemic and Critically Endangered Mole Salamander (*Ambystoma leorae*) of Central Mexico

**DOI:** 10.1371/journal.pone.0103595

**Published:** 2014-07-30

**Authors:** Armando Sunny, Octavio Monroy-Vilchis, Carlos Reyna-Valencia, Martha M. Zarco-González

**Affiliations:** Estación Biológica Sierra Nanchititla, Facultad de Ciencias, Universidad Autónoma del Estado de México, Toluca, Estado de México, México; Institute of Biochemistry and Biology, Germany

## Abstract

The reduced immigration and emigration rates resulting from the lack of landscape connectivity of patches and the hospitality of the intervening matrix could favor the loss of alleles through genetic drift and an increased chance of inbreeding. In order for isolated populations to maintain sufficient levels of genetic diversity and adapt to environmental changes, one important conservation goal must be to preserve or reestablish connectivity among patches in a fragmented landscape. We studied the last known population of *Ambystoma leorae,* an endemic and critically threatened species. The aims of this study were: (1) to assess the demographic parameters of *A. leorae* and to distinguish and characterize the microhabitats in the river, (2) to determine the number of existing genetic groups or demes of *A. leorae* and to describe possible relationships between microhabitats types and demes, (3) to determine gene flow between demes, and (4) to search for geographic locations of genetic discontinuities that limit gene flow between demes. We found three types of microhabitats and three genetically differentiated subpopulations with a significant level of genetic structure. In addition, we found slight genetic barriers. Our results suggest that mole salamander’s species are very sensitive to microhabitat features and relatively narrow obstacles in their path. The estimates of bidirectional gene flow are consistent with the pattern of a stepping stone model between demes, where migration occurs between adjacent demes, but there is low gene flow between distant demes. We can also conclude that there is a positive correlation between microhabitats and genetic structure in this population.

## Introduction

Habitat loss is the leading cause of species declines and extinctions worldwide [1]. Habitat fragmentation by human activity poses further problems for species already in decline. Small habitat patches may contain small populations isolated from conspecifics that are unreachable due to migration barriers. These populations are at great extinction risk at the hand of either stochastic or further deterministic causes, as their genetic diversity and subsequent biological fitness is reduced over time. Amphibians often have patchy distributions due to habitat specificity and strict ecophysiological requirements [2], [3] and naturally exhibit metapopulation structure [4]–[7]. The reduced immigration and emigration rates resulting from the lack of landscape connectivity between patches and the hospitality of the intervening matrix could favor the loss of alleles through genetic drift and an increased chance of inbreeding.

In order for fragmented populations to maintain sufficient levels of genetic diversity, one important conservation goal must be to preserve or reestablish landscape connectivity among patches. This may involve eliminating migration barriers, providing migration corridors between patches, and even manually moving individuals as a last resort [8], [9]. Populations found to be connected by gene flow, should be managed as a single unit in order to maintain migration in the future [10]. Another conservation strategy that can avoid isolation is promoting larger areas of habitat and protecting groups of patches in close proximity. Larger habitat patches should support larger populations, which can defend against drift and stochastic extinctions. A key to conserving fragmented populations is addressing the spatial scale at which a given species is negatively affected by fragmentation [11]. The spatial scale varies depending on the species migratory ability through the intervening matrix and on its population size within the patches. To effectively conserve amphibian biodiversity and make informed management decisions, we need to understand their current population structure, genetic diversity, configuration of habitat patches, and life histories [12], [13].

*Ambystoma leorae* [14] is a micro-endemic mole salamander from Sierra Nevada, Central Mexico. The only known remaining population is located in two small rivers of approximately 1 km. [15]. This neotenic species is restricted to mountain streams (2 m wide×0.5 m depth) with cold water temperature (12 to 15°C), in *Abies religiosa* forest Information about their reproduction is limited and only one pregnant salamander has been found [17]–[19]. Because of the species’ limited distribution, the clearance of the forest, and the pollution and consumption of the water by humans, it is classified as a critically endangered species [20] and as a threatened species by the Mexican government [21]. In the geographic distribution of the species, there are several threats that are modifying the ecosystem, including alteration of the stream to collect the water for human consumption, introduction of cattle, and the direct collection of specimens for traditional food. Ecological and genetic studies are important in order to provide information to make management decisions and to preserve this species.

The aims of this study are: (1) to assess the demographic parameters of *A. leorae* and to distinguish and characterize the microhabitats in the river, (2) to determine the number of existing genetic groups or demes of *A. leorae* and to describe possible relationships between microhabitats types and demes, (3) to determine gene flow between demes, and (4) to search for geographic locations of genetic discontinuities that limit gene flow between demes.

## Materials and Methods

The manipulation of mole salamanders was conducted with permits received for field work from the Mexican government through to the Ministry of Environment and Natural Resources (SEMARNAT; permit SGPA/DGVS/05457/13). Our study received the approval of the ethics committee of Universidad Autónoma del Estado de México.

### Study area

**Figure 1:**
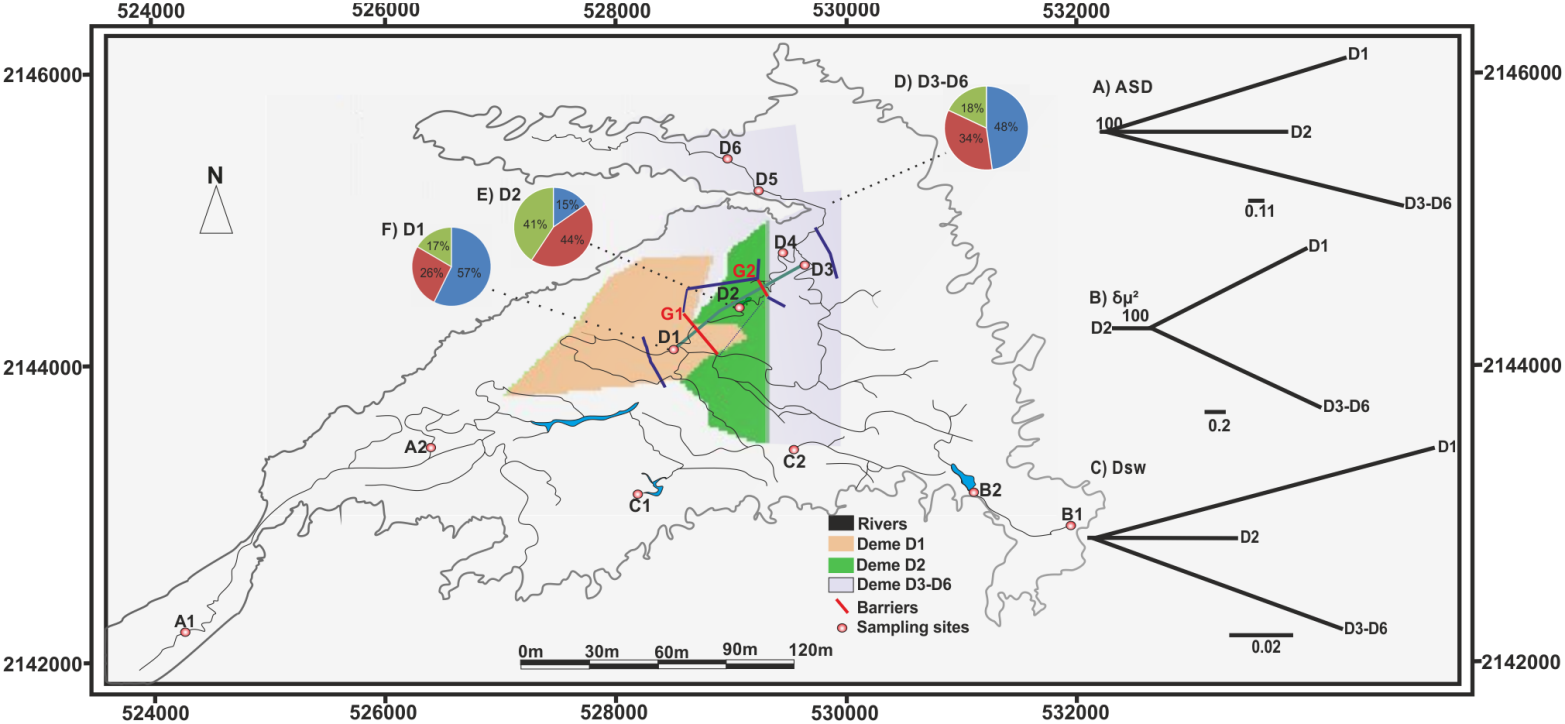
Study site map with sampling locations (A1–D6). The geographical representation of the genetic clusters obtained by GENELAND are: orange-D1, green-D2 and white-D3–D6). The red lines G1 and G2 are genetic barriers found with the software BARRIER. Neighbor-joining (NJ) trees constructed using A) ASD (Goldstein et al. 1995a), B) δµ^2^ (Goldstein et al. 1995b) and C) D_SW_ (Shriver et al. 1995) display genetic distances. Bootstrap values are shown along branches and pie chart colors represents the proportions of the three genetic demes assigned by the software STRUCTURE in each deme.

Iztaccihuatl-Popocatepetl National Park (IPNP) is located in Central Mexico, near Mexico City, The geographical coordinates are 19°21′09″N, 98°40′11″W and 19°35′25″N, 98°66′97″ W, with an altitude of 4,130 masl (Fig. 1). Sampling was in two tributaries separated by 0.56 km that flow into the same river after 0.68 km considering a linear distance from the spring of the tributaries. These tributaries flow over plains and gentle slopes, with pools approximately 5 m from each other. The principal vegetation type outside the tributaries are small alpine grassland (*Muhlenbergia sp*.) surrounded by forest (*Pinus hartwegii* and *Abies religiosa*).

### Population sampling

Sampling effort per visit was 4 persons for 4.5 hours per day for 2 days per month for 8 months. Each mole salamander encountered was measured (both snout vent length (SVL) and total length (TL)) and weighed. We identified age class based on size: individuals were classified as egg, juvenile (gilled larva), adult or metamorph, according the Sturgess rule [22]. Exact collection localities were determined using a global positioning system and the microhabitat where the individual was found was noted. To compare the observed abundance between sites, we calculated the rate of encounter [23].

### Habitat description

Nine microhabitat characteristics were recorded within a quadrat of 10 m^2^ at all twelve sampling sites defined a priori based on differences in the percentage of cover vegetation and type of substrates (stones, sand and mud). These characteristics included percentage area covered by grass bushes outside the river (COHAB1), percentage area covered by *Pinus*-*Abies* forest outside the river (COHERB2), percentage area covered by vegetation inside the river (COVEGR), percentage area covered by stones in the river (COSTONR), percentage area covered by mud (COMUDR), depth of the river (DEPR), width of river (WIDER), temperature outside of the river (TEMPOUTR), and river water temperature (TEMPR). To reduce the number of microhabitat variables, we performed a principal components analysis (PCA) using the software STATISTICA 8.0 [24]. Using the most important variables found in the PCA, we created a Ward’s dendogram [25], to determine the micohabitat types.

### Genetic data collection and microsatellite typing

A combination of adult and neotenic larval salamanders were included in this study. We extracted genomic DNA from tissue samples (tail clip) from 96 individuals of *Ambystoma leorae*, from six sample sites, using QIAGEN extraction kits (Qiagen Inc., Valencia, CA, USA) for use as templates in PCR amplification of nine microsatellite loci following published protocols [26]. Loci were multiplexed and fragment sizes determined using sized Rox-500 standards. Results were scored using GENEMAPPER 4.0 (Applied Biosystems). We measured fragment lengths and binned with TANDEM 1.08 [27]. Multiple samples were sized at least twice to assure reproducibility and correct readings. We used GIMLET 1.3.2 [28] to identify samples that were duplicate genotypes and removed them from the analysis. To test for null alleles and large allele dropout we used MICROCHECKER [29].

### Genetic diversity

We calculated genetic diversity indices (number of alleles, effective number of alleles, and number of private alleles) using the software GENALEX 6 [30], POPGENE 1.31 [31] and GENETIC STUDIO 0.1 [32]. Expected and observed heterozygosities, departures from Hardy–Weinberg equilibrium, and linkage disequilibrium are described in Sunny *et al.* [7]. In addition, we analysed whether the latitude influenced the observed and expected heterozygosity and F_is_ using GENETIC STUDIO. Finally, to determine if some demographic parameters and habitat characteristics were linked to the percentage of alleles and genotypes, we completed a multivariate analysis of Sperman’s correlation [33], with a 95% confidence level.

### Population genetic structure

For population sampling, we defined twelve microhabitat types a priori based on differences in the percentage of cover vegetation and type of substrates (stones, sand and mud). (Fig. 1, A1 to D6). We applied an individual-based method to investigate the genetic structure based on three Bayesian clustering analyses. The first analysis used STRUCTURE 2.3.3 [34]–[36]. The number of genetic groups (K) was determined testing the K value from 1 to 10 and running the analysis ten times per K value in order to determine the maximum value of posterior likelihood [lnP(D)]. Each run was performed using 1000000 burn in periods and 1000000 MCMC iterations, with a correlated allele frequencies admixture model and without prior information on population origin. We determined the most probable ΔK value using the maximum value of DK, following the method of Evanno *et al.* [36] using the software STRUCTURE HARVESTER [38]. In the second Bayesian clustering analysis, we used the software GENELAND 3.2.2 [39]. We assumed a correlated allelic frequencies model and a true spatial model [39], with a coordinate uncertainty value of 100 m. We performed ten independent runs with 1000000 iterations, 100 thinning and 100 burn in, ranging from 1 to 10. When we got the maximum number of possible genetic groups, we proceeded with the assigning of individuals, using twenty independent runs with 1000000 iterations, 100 thinning and 1000 burn in. The third method used was also a Bayesian clustering algorithm that included spatial information in the form of hidden Markov random fields [40] implemented in TESS 2.3 [41]. We used the F model and admixture with 50000 simulations (10000 burn-in) to estimate K and to assign individuals to clusters. We chose 50000 simulations because convergence was always reached at this level after five independent runs [42]. In the software GENETIC STUDIO and GENEPOP 3.4 [43], we calculate the F_st_, R_st_ and D_est_ with 10000 dememorization steps and 1000 batches of 10000 iterations per batch in order to test the divergence between demes. For the latter we estimated different genetic distances in accordance with a SMM mutation model: ASD [44], δµ^2^ [45] and D_SW_ [46]. Neighbor-Joining (NJ) trees with 1,000 replicates to estimate bootstrap values were constructed with the software POPULATIONS 1.2.30 [47]. These distances then were used to construct NJ trees to cluster individuals by genetic similarity using the software FIGTREE 1.3.1 [48].

### Barriers analysis

We used the software BARRIER 2.2 [49] to find the geographic locations of genetic discontinuities among *A. leorae* demes. This software implements Monmonier’s maximum difference algorithm to reveal the genetic discrepancies associated with the maximum values of a genetic distance matrix. We used a pairwise matrix of Nei’s genetic distances [50] estimated for the three demes found. We resampled random subsets of the individuals within the software MSANALYSER 4.05 [51] which provided 100 bootstrap replicate distances that were used to achieve statistical significance for the predicted barriers.

### Migration and isolation by distance

Gene flow between demes was estimated using the Bayesian inference implemented in MIGRATE-n 3.0 [52]–[54]. We used a Brownian approximation model, the parameters estimated with this software were five independent runs using four longs chains with a run of 1×10^7^ recorded genealogies sampled every 1000 steps and a burn in of 1×10^6^. Four hot chains were used with temperatures: T1 = 1.0, T2 = 1.5, T3 = 3.0 and T4 = 1.0×10^6^. We used default values for the remaining parameters. To estimate the number of migrants per generation (Nem) we multiply M by θ. We tested four hypotheses of migration among groups: (1) migration to and from all groups, (2) migration only between adjacent groups, (3) migration between distant groups, and (4) all groups as part of a panmitic population. The same parameters were used for all models. To compare the models and choose the most likely model, we used the logarithm of the Bayes Factor (LBF, 53). We also made a skyline plot in order to visualize the changes of the population sizes and migration rates through time [55]. Using the software GENETIC STUDIO, we tested the correlation between the pairwise genetic and geographical distance between demes by short or long distance dispersal events using the Graph Theory [56]. This test examines how genetic variation is distributed across the landscape [32].

## Results

### Population sampling

**Figure 2:**
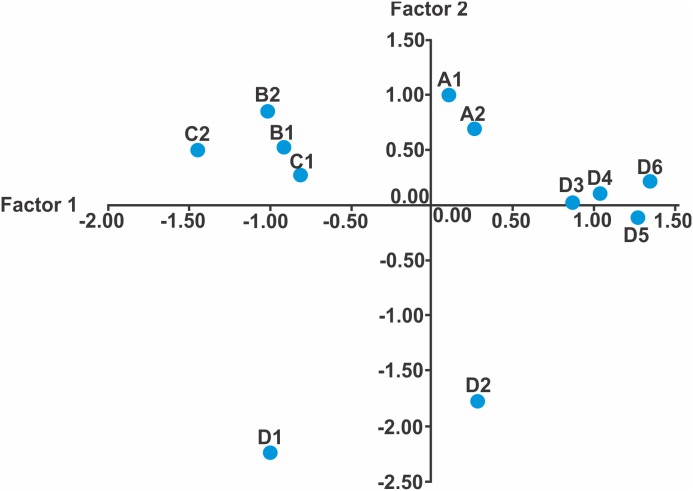
PCA analysis. Displays factor 1 (percentage of water without vegetation and the percentage of water with vegetation and stones) and factor 2 (percentage of grass bushes outside the river and the percentage of the pine-oak forest outside the river). The characteristics of the sites are: A1, A2 = COHAB1 100% COHERB2 0%, COVEGR 28%, COSTONR 28%, B1–C2 = COHAB197%, COHERB2 3%, COVEGR88%, COSTONR 7%, D1 = COHAB150%, COHERB240%, COVEGR 40%, COSTONR 15%, D2 = COHAB1 65%, COHERB2 40%, COVEGR 28%, COSTONR 65%, and D3–D6 = COHAB 83%, COHERB27%, COVEGR 0%, COSTONR 88%.

We recorded 161 mole salamanders in 2011 (Fig. S1), *A. leorae* was in seven of the twelve sampling sites. The mean rate of encounter in the seven sites was 2.08 mole salamanders per visit. We found 44 eggs distributed in 11 clutches (min 1, max 10 eggs) from February to June. The clutches were attached to aquatic vegetation and inside caves. The egg shape was almost round and its size was variable (8.5 to 20 mm). One hundred fifty two gilled larva were found. These were classified into eight age classes: (1) 42 to 65 mm, (2) 65.1 to 88 mm, (3) 88.1 to 111 mm, (4) 111.1 to 134 mm, (5) 134.1 to 157 mm, (6) 157.1 to 180 mm, (7) 180.1 to 203 mm, and (8) 203.1 to 226 mm. Class nine included transformed mole salamanders (Fig. 2). The maximum number of individuals was found in July (N = 43) and the minimum number of individuals was found in August (N = 2) (Fig. S1). The maximum number of mole salamanders was found at site D3–D6 (N = 79) while just one mole salamander was found at site A1 (this individual was excluded from further genetic analysis because of the low sample size) (Fig. S2).

### Habitat description

The sites with *A. leorae* had the following characteristics: small pools with sandy, muddy or rocky bottoms (0.033–0.47 m depth and 1.35–3.75 m width), slow stream (0.3–0.4 m/s), water temperature that ranged from 12 to 18.5°C, water with pH from 6.9 to 7.4, and high water oxygen levels of 78% concentration (6.25 mg/L). The sites had different percentages of aquatic plants ranging from 0% to 40%. Outside the rivers the vegetation was mainly small alpine grassland (*Muhlenbergia sp*.) surrounded by forest (*Pinus hartwegii* and *Abies religiosa*). Air temperature ranged from 10 to 12.5°C (Table S4). All sites had signs of human disturbance, such as garbage and cattle presence.

### PCA and cluster analysis

**Figure 3:**
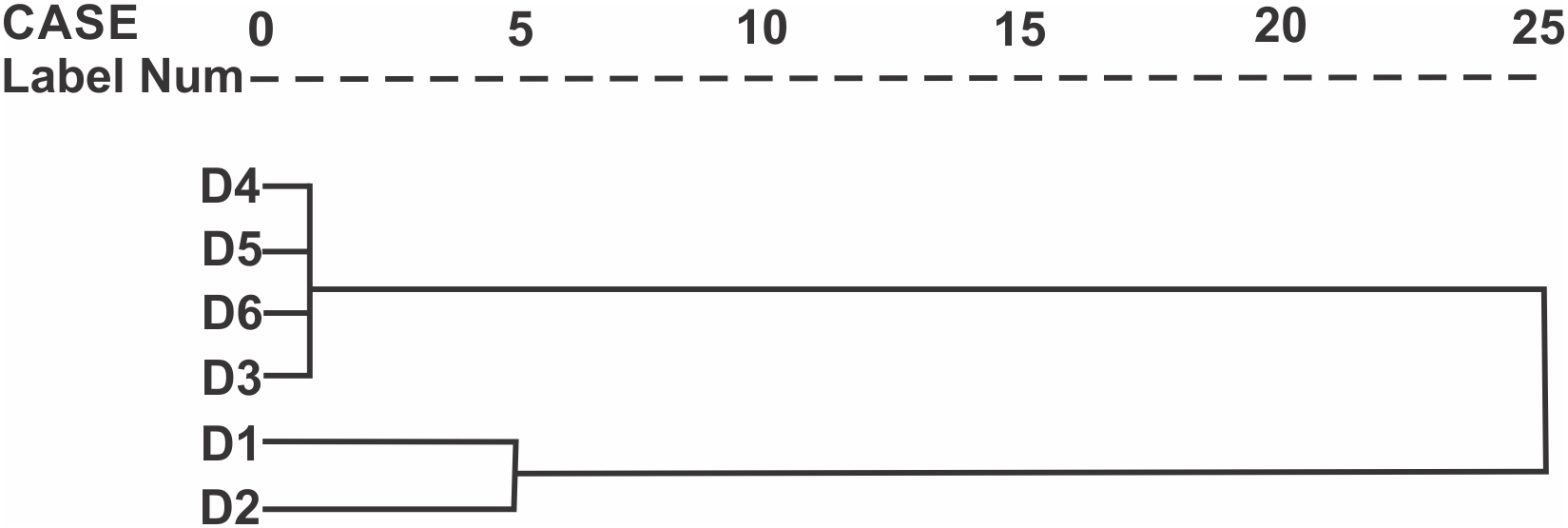
Dendogram using the Ward’s method. Showing three types of microhabitat (D1, D2 and D3–D6), the microhabitat D1 and D2 are more similar between them and D3 to D6 are the same type of microhabitat.

The first three factors explained 78.6% of the variation (Table S1). The first factor was associated with the percentage of water without vegetation and the percentage of water with vegetation and stones (Table S2 and Fig. 2). The second factor was associated with the percentage of grass bushes outside the river and the percentage of the *Pinus*-*Abies* forest outside the river. The third factor was associated with the water temperature (Table S2 and Fig. 2). The dendogram found three types of microhabitats or clusters D1, D2 and D3–D6 (Fig. 3). The first cluster was the deme D1 with the following characteristics: COHAB1 = 50% COHERB2 = 40%, COVEGR = 40%, COSTONR = 15%. The second cluster included the second deme D2 with the following characteristics: COHAB1 = 65%, COHERB2 = 40%, COVEGR = 28%, COSTONR 65%. Demes D3, D4, D5, and D6 comprised the third cluster with: COHAB1 = 83%, COHERB2 = 7%, COVEGR = 0%, COSTONR  = 88%. We defined these populations as D3–D6. The characteristics of the A1–A2 sites with only one individual were: COHAB1 = 100%, COHERB2 = 0%, COVEGR = 28%, COSTONR = 28%. The sites without individuals had the following characteristics (B1 though C2): COHAB1 = 97%, COHERB2 = 3%, COVEGR = 88%, COSTONR = 7%.

### Microsatellite typing and population genetic structure

**Figure 4:**
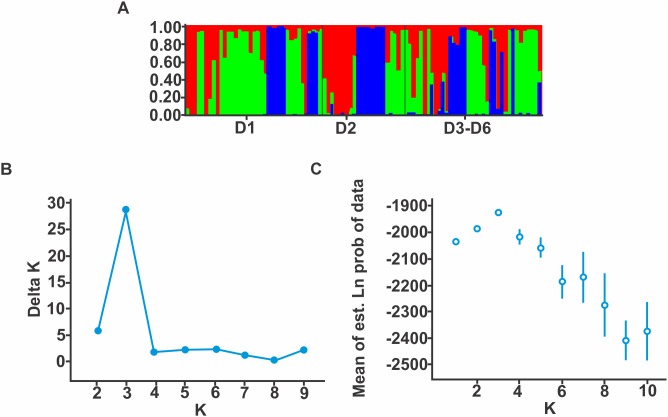
Population genetic structure of *A. leorae* analyzed with STRUCTURE. Each individual is represented by a horizontal line that is partitioned into K components representing the ancestry fractions in K = 3 clusters. Population and black lines separate individuals order individuals from different population. B) and C) Evanno et al. (2005) plots for detecting the number of K groups that best fit the data.

All samples were polymorphic at all loci, we did not detect recaptured individuals. No large allele dropout or null alleles were detected. The three Bayesian clustering methods detected three genetic groups. STRUCTURE (LnPr (k = 3) = –1911.4) (Fig. 1, F, D, E pie charts and Fig. 4) and ΔK method confirmed this result (Fig. 4B, 4C and Table S3). GENELAND (Fig. 1, green, orange and white shades) and TESS ((K = 3 with a DIC value = 3757.21)). GENELAND and STRUCTURE assigned the same individuals to the same genetic group with a 100% of similarity. The F_ST_, R_ST_ and D_est_ divergences was low between demes (Table 1). Genetic structuring was low among demes (F_ST_ = 0.021, R_ST_ = 0.031 and D_est_ = 0.007) and the effective number of genetically distinct groups according Jost [56] calculated for all loci was 1.038 (Table 2). The NJ trees showed two topologies. The first was inferred with the δµ^2^ mutation model. The population D1 and D3–D6 were sister groups and D2 was the ancestral deme. However, the other two analyses (ASD and D_SW_) showed an alternative topology in which D1 and D2 were sister groups and D3–D6 was the ancestral deme. (Fig. 1, NJ trees A, B and C).

**Table 1:** Genetic differentiation for each deme. Below the diagonal F_ST_ and (R_ST_), above the diagonal D_est_.

	D1	D2	D3–D6
**D1**	**-**	0.013	0.004
**D2**	0.031 (0.015)	**-**	0.004
**D3–D6**	0.017 (0.060)	0.016 (0.016)	**-**

**Table 2:** ΔS component of the genetic diversity and effective number of distinct genetic groups according to Jost (2008).

Locus	Δ_ST_
Atig52.143	1.018
Atig52.115	1.029
At60.3	1.005
At52.1	1.044
At52.2	1.048
A52.6	1.008
At52.20	1.062
At52.10	1.062
At52.34	1.068
**Mean**	**1.038**

### Genetic diversity

**Figure 5:**
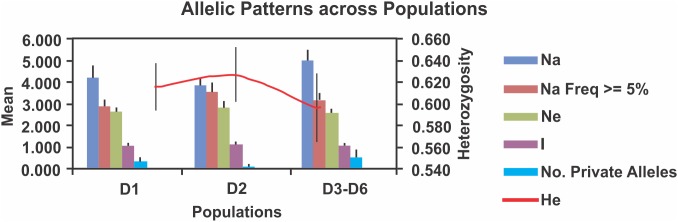
Patterns of allelic richness and heterozygosity in the three demes. Na = Number of different alleles, Na (Freq > = 5%) = Number of different alleles with a frequency > = 5%, Ne = Number of effective alleles = 1/(Sum pi∧2), I = Shannon’s information index, Number of private alleles = Number of alleles unique to a single population, He = Expected heterozygosity.

Forty nine alleles were found in all demes. The mean observed alleles was 4.3 for deme D1, 3.9 for deme D2, and 5.0 for demes D3–D6 (Fig. 5). For all demes, 67 genotypes were found. The mean genotype was 5.3 for deme D1, 4.3 for deme D2, and 5.3 for demes D3–D6. We found a positive correlation between the latitude and the Ho and He. As the latitude increased, the observed and expected heterozygosity decreased, while the F_is_ increased (Fig. S5). We found a positive relationship between percentage of alleles and genotypes and percentage of neotienic juveniles classes type 4 to 8 (Spearman = 1, p = 0). In addition, a negative correlation was found between percentage of alleles and genotypes and percentage of juveniles classes type 1 to 3 (Spearman = −1, p = 0) and percentage of transformed mole salamanders (Spearman = −1, p = 0) (Table. 3). Finally, no statistical relationship was found between the percentage of alleles and genotypes and the microhabitat characteristics. We only found a positive tendency between percentage of alleles and genotypes with COHAB1 (Spearman = 0.866, p = 0.22).

**Table 3:** Multivariate analysis of relationships between the demographic parameters, the habitat characteristics and the percentage of alleles and genotypes.

	Percentage of juveniles(classes type 1 to 3)	Percentage oftransformedmole salamanders	Percentage of neotenicjuveniles (classes type4 to 8)	COHAB1	COHERB2	COVEGR	COSTONR
Percentage of alleles andgenotypes	–1 (0)	–1 (0)	1 (0)	0.866 (0.221)	–0.5 (0.479)	0.5 (0.479)	–0.5 (0.479)

### Barriers detected

The genetic barriers detected by the Monmonier’s maximum difference algorithm separated deme D2 from the other two demes (D1, D3–D6) assigned by STRUCTURE, GENELAND and TESS (G1, G2 in Fig. 1). Similar results exist with the Poisson-Voronoi tessellation model implemented in GENELAND (shaded orange, green, and white in Fig. 1, red lines represent barriers, G1 and G2).

### Migration and isolation by distance

Isolation by geographic distance was not found (Z = −49143.548, p = 0.602; Fig. S4). There was a greater amount of short nodes indicating that most migration occurred between adjacent demes. However, there were also long nodes showing that there was less migration between distant locations. (Fig. S4). The best model found with the Beizer estimation was migration between neighborhoods (LBF = −0.190; Table 4). All migration models had M values greater than one, indicating that migration (and not mutation) was the main factor contributing to the genetic variation in these groups. The estimates of θ for the three demes were: D1 = 0.097, D2 = 0.097 and D3–D6 = 0.098. The demes that had the highest number of migrants per generation were D3–D6 to D2 = 9.3. The lowest number of migrants was between D1 to D3–D6 = 3.5 (Table 5). The Bayesian skyline plot indicated that the population has undergone several expansions and declines, showing a multimodal model pattern (Fig. S6).

**Table 4:** Support for the migration model between demes.

	Bezier	Bezier	Bezier
Migration models	lmL	LBF	BF
Migration between neighborhoods	**–152373.140**	**–0.190**	**0.909**
Migration between distant demes	–205931.710	0.412	1.229
Panmitic population	–256528.930	0.852	1.531

**Table 5:** Asymmetric immigration rates inferred in MIGRATE between demes.

	D1	D2	D3–D6
**D1**	**-**	3.9	3.5
**D2**	4.1	**-**	4.6
**D3–D6**	3.7	9.3	**-**

## Discussion

In the present study, we focused on a critically endangered mole salamander and we obtained the largest number of samples of this species ever recorded (N = 161). Historical studies have found significantly less individuals. In 1943, only four individuals were found at the type locality Río Frío town [14]. In 1985, Lemos-Espinal and Amaya-Elias [1985] located six individuals in an eight-year study in the river Coatzala. Later Vega-Lopez and Alvarez [18] found ten individuals on three different locations, and finally Lemos-Espinal *et al.* [19] found 59 individuals in the river Tonatzin. In fact, Casas-Andreu [58] states that *A. leorae* has not been deposited in scientific collections since 1973. In all previous studies, little is mentioned about reproduction. Only Vega-López and Alvarez [18] found a female with 2.5 mm eggs in June. In this study, for the first time, we described the clutches and egg size. *A. leorae* clutches are similar in size to those of *A. altamirani* [59] and significantly lower than those of other mole salamander’s species, where clutch size reached 77 to 1,691 eggs per clutch [60]–[64]. This may be because these species, especially *A. mexicanum* and *A. lermaense,* have a greater average snout vent length than *A. leorae.* In addition, the egg size is considerably larger than those of other species of mole salamanders [59], [62], [63]. This is important to consider because a positive relationship exists between the size of the female and the number of eggs per clutch in reptiles and amphibians [63], [65]–[68]. Furthermore, amphibians in high altitudes and latitudes tend to have a low production of eggs [69]. The low egg production may also be due to features of *A. leorae*’s habitat such as limited availability of resources and reduced predation, compared to the environments of *A. mexicanum* and *A. lermaense*’s [70].

Most individuals in this study were collected from March to July (N = 139, Fig. S1) and the majority were gilled larva of class type 5 (Fig. S3). This species, like other mole salamanders, exhibits neoteny, meaning they reach sexual maturity without undergoing metamorphosis. Huacuz [71] reported neotenic males of *A. rivulare* with SVL ranging from 60 to 90 mm and 40 to 100 mm for females. Additional studies have found indiviuals of *A. dumerilii* with SVL of 122 mm [72], [73], *A. altamirani* with SVL average of 64 mm [74]. *A. ordinarium* with SVL average of 56 mm [75], and for *A. tigrinum tigrinum* with average of 94 mm and minimum 80 mm [76]. Based on these previous findings, we assume that *A. leorae* becomes sexually mature once they reached the class type 4. Juveniles of the class types 1 to 3 possibly hatched in late January and early February. In other mole salamander, the period of incubation has been found to occur between August and December [59], [77] with a one month to 55-day incubation period [78], [79]. The low capture success with the 2 and 3 class type of gilled larva in this study may be due to the fact that at this early stage the gilled larva are in refuges inside caves. In addition, mortality at this stage is very high due to predation, disease, changes in temperature ranges, and cannibalism [79]–[82].

### Microenvironment

Microhabitat characteristics for *A. leorae* have not previously been described in detail. There are only scare reports on water temperature [17]–[19] and vegetation type around streams [19]. We found average water temperature to be six degrees lower than previous reports [19]. There has been no description of the percentage of water oxygenation provided in the literature. We found that highly elevated oxygenation water level (78% dissolved oxygen) are necessary for the mole salamander survival. In sampling zones with low oxygenation water (B1, B2, C1 and C2) we did not find individuals.

For population sampling, we defined twelve microhabitat types a priori; however, after the principal component analysis and the Ward’s dendogram, we found only three different microhabitats types (Fig. 3). The variables generating the differentiation between microhabitats types were: percentage area covered by grass bushes outside the river, percentage area covered by *Pinus*-*Abies* forest outside the river, percentage area covered by vegetation inside the river and percentage area covered by stones inside the river. The mole salamanders need certain habitat characteristics in order to survive, grow, and reproduce. We believe the percentage area covered by grass bushes outside the river may help to avoid water heating and to decrease predation risk [83–87). The percentage area covered by *Pinus*-*Abies* forest outside the river is important because the salamander presence and abundance are highly correlated with forest cover [88]–[90], and dispersion occurs through the forest [91], [92]. In addition, some amount of vegetation cover inside the river is needed for ovoposition [93]–[95], as most clutches found were attached to vegetation. The vegetation complexity within a pond might also enhance habitat quality [96], [93], [94]. Finally, stones provide an important refuge for individuals to hide from predators (pers. obs.). We found a negative relationship between the gilled larvas of class type 1 to 3, this is due they being newly hatched individuals possibly close related individuals. However, the relationship is positive between the neotenic gilled larva of classes 4 to 8, most of this reproductive individuals are not strongly related as describe Sunny *et al.* [7]. In addition, we found a positive relationship between the number of alleles and genotypes in places with a major percentage area covered by grass bushes outside the river. For future studies, we recommend increasing the sampling localities and labeling individuals to determine dispersal patterns and survival rates in order to better understand the demographics of the species and to inform management and conservation plans.

### Genetic structure

We found within-population structure; which is not surprising for this species due to its known high breeding site fidelity and low dispersal capability. This within-population structure can also arise due to the existence of temporal reproductive cohorts [97]. Mole salamanders, with a mating system in which adults congregate in a pool to breed [98], [99], may exhibit some level of population structure within a larger metapopulation [3], [7], [87]. Some populations are separated by environmental characteristics and should display certain genetic structure, especially if the effective population size is small. In this study, we detected low significant genetic differentiation between the three demes, but sufficient levels to separate the metapopulation. The metapopulation structure finding and the F_ST_ values are similar to those detected in other studies of mole salamander’s population genetics in fragmented habitats [97], [100], [101]. The results suggest that a combination of the characteristics of the microhabitats and river constrictions between D1 and D2 and between D2 and D3–D6 demes may be generating the three described demes. Ours results suggest that mole salamander species are very sensitive to microhabitat features and even relatively narrow obstacles in their path. The estimates of bidirectional gene flow is consistent with the pattern of a stepping stone model where migration occurs between adjacent demes, but there is low gene flow between distant demes. We propose that connectivity should be reestablished among these three demes, eliminating the detected migration barriers to avoid the loss of alleles through genetic drift and inbreeding of this metapopulation.

## Supporting Information

Figure S1 **Individuals collected per month.**
(TIF)

Figure S2 **Individuals per class in each deme.**
(TIF)

Figure S3 **Classes obtained from Sturges rule (1926) and individuals per class.**
(TIF)

Figure S4 **Matrix scatter plot of physical distance as a function of genetic distance.**
(TIF)

Figure S5 **Diversity gradient plot showing observed heterozygosity (HO), expected heterozygosity (HE), expected heterozygosity corrected for small sample sizes (HE), and inbreeding (FIS).**
(TIF)

Figure S6 **Skyline plot of a population that recently increased strongly, the time is in units of mutation scaled generations.**
(TIF)

Table S1 **Eigenvalues of each factor and the cumulative score of each factor. In bold are the most important values.**
(DOCX)

Table S2 **Scores of each microhabitat characteristics of the factor analysis components. In bold are the most important variables.**
(DOCX)

Table S3 **Table output of the Evanno method results. In bold is the largest value in the Delta K column.**
(DOCX)

Table S4 **Microenvironmental conditions range.**
(DOCX)
