# Supplementary figures and images for: Microhabitat Types Promote the Genetic Structure of a Micro-Endemic and Critically Endangered Mole Salamander (Ambystoma leorae) of Central Mexico
Source: PLoS One. 2014 Jul 30;9(7):e103595. doi: 10.1371/journal.pone.0103595 (PMC4116214; doi:10.1371/journal.pone.0103595)

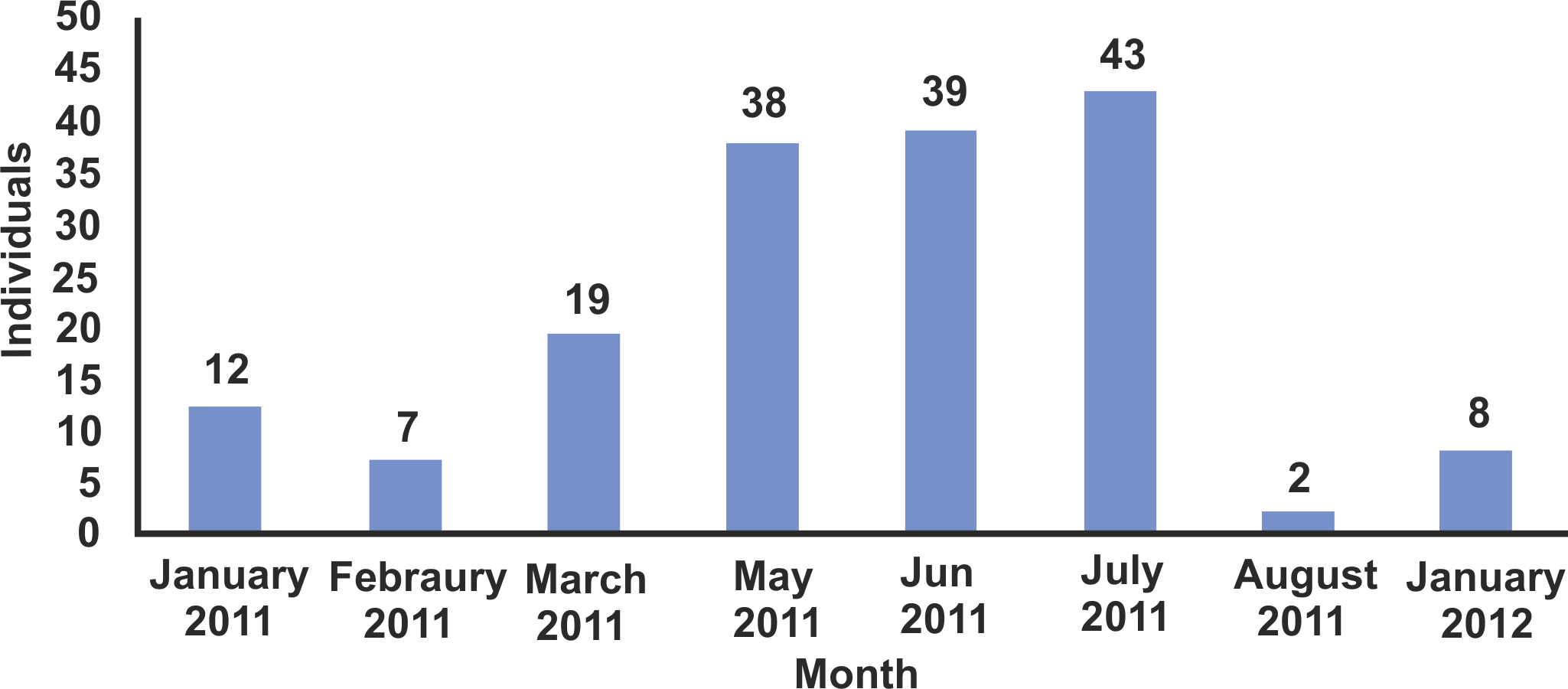

Supplement: Figure S1 — Individuals collected per month. (TIF) [file pone.0103595.s001.tif]

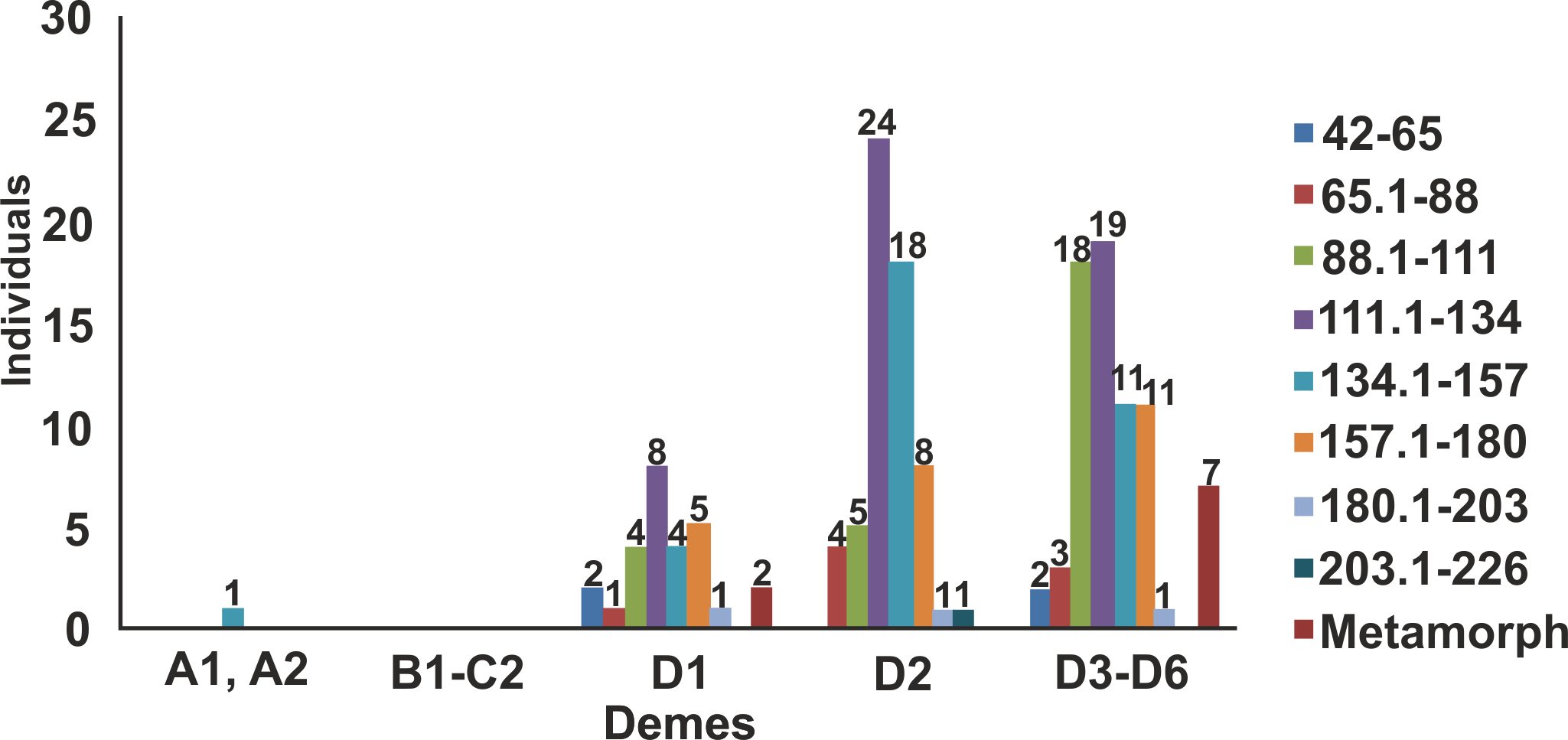

Supplement: Figure S2 — Individuals per class in each deme. (TIF) [file pone.0103595.s002.tif]

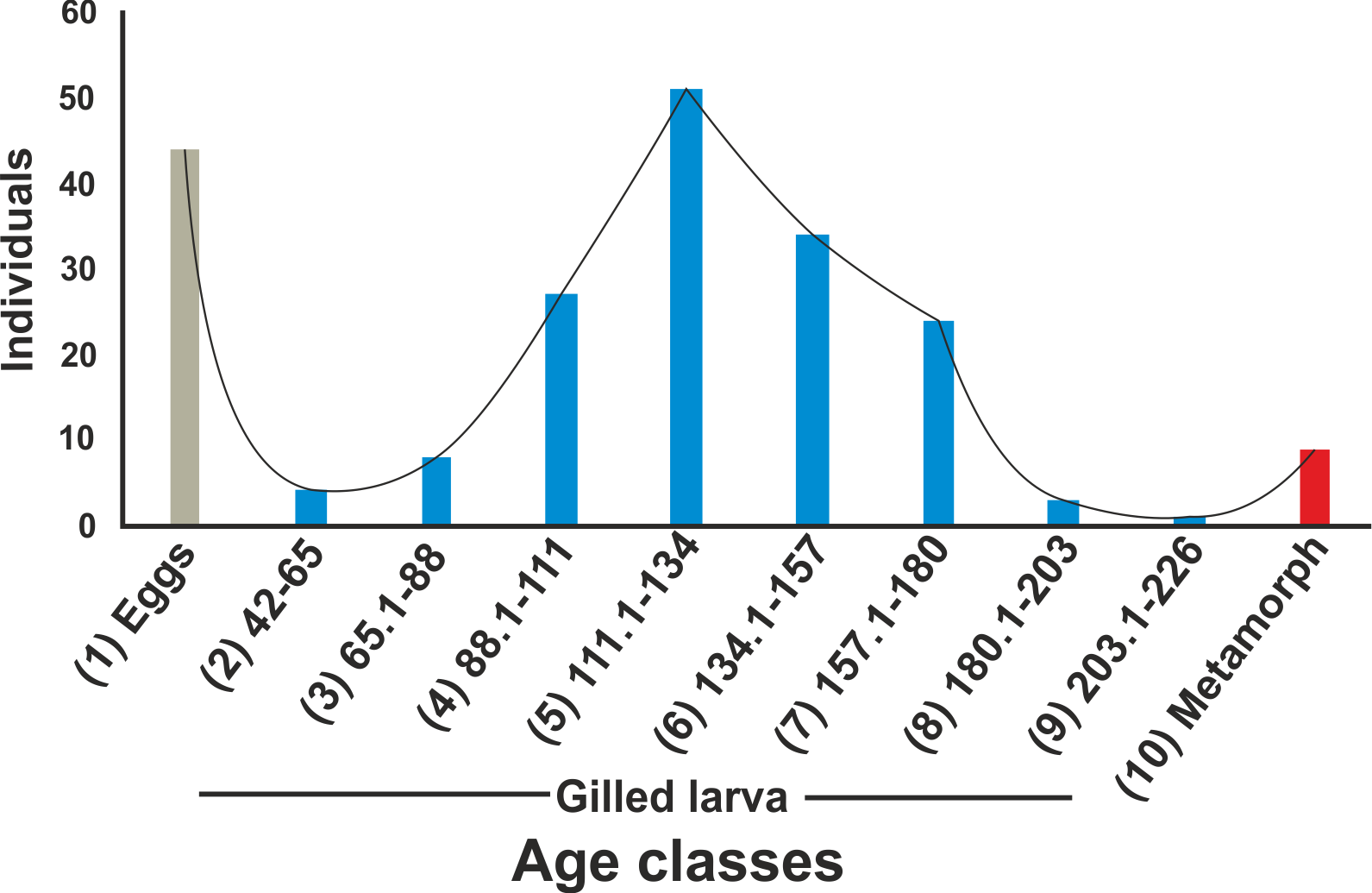

Supplement: Figure S3 — Classes obtained from Sturges rule (1926) and individuals per class. (TIF) [file pone.0103595.s003.tif]

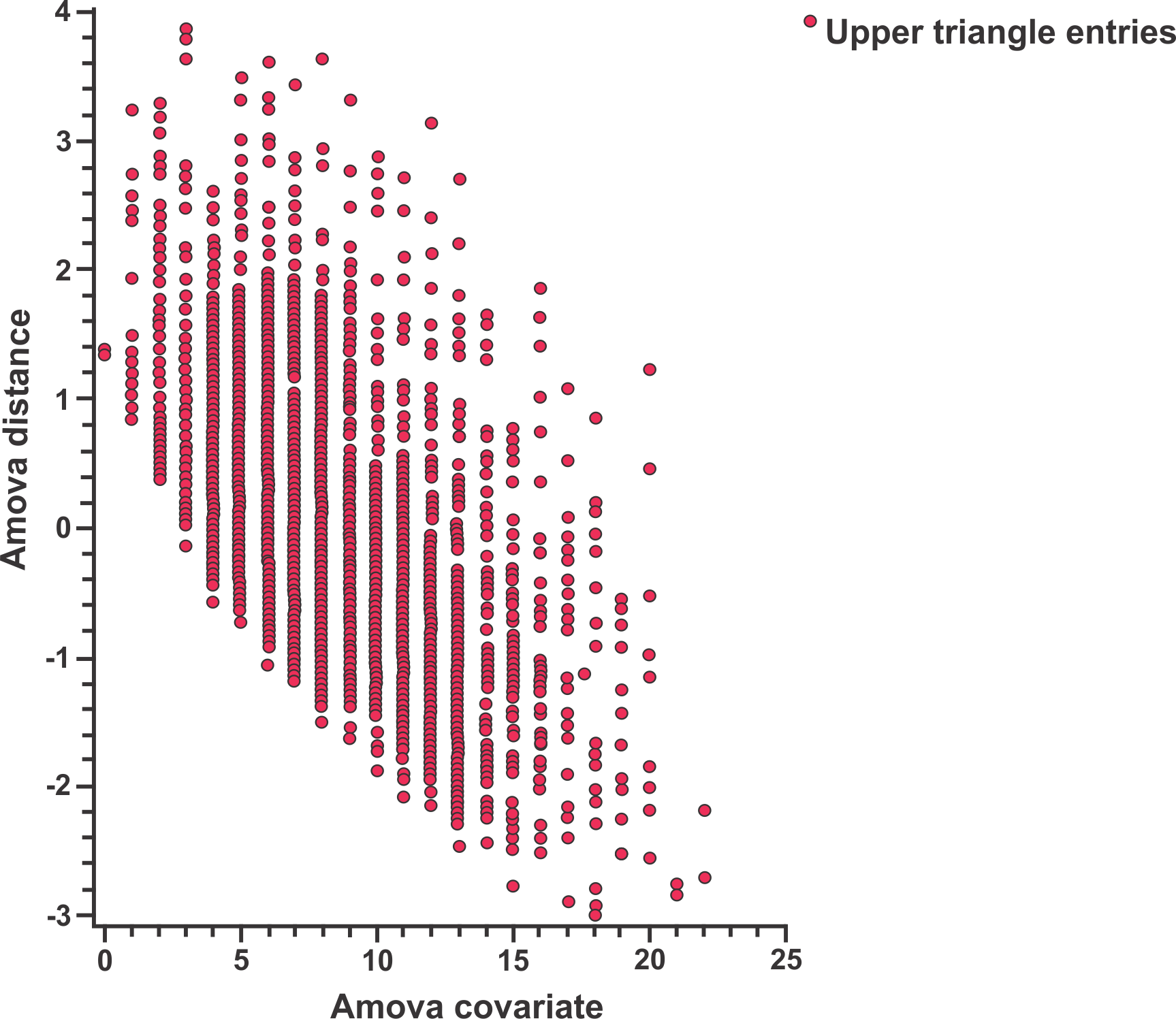

Supplement: Figure S4 — Matrix scatter plot of physical distance as a function of genetic distance. (TIF) [file pone.0103595.s004.tif]

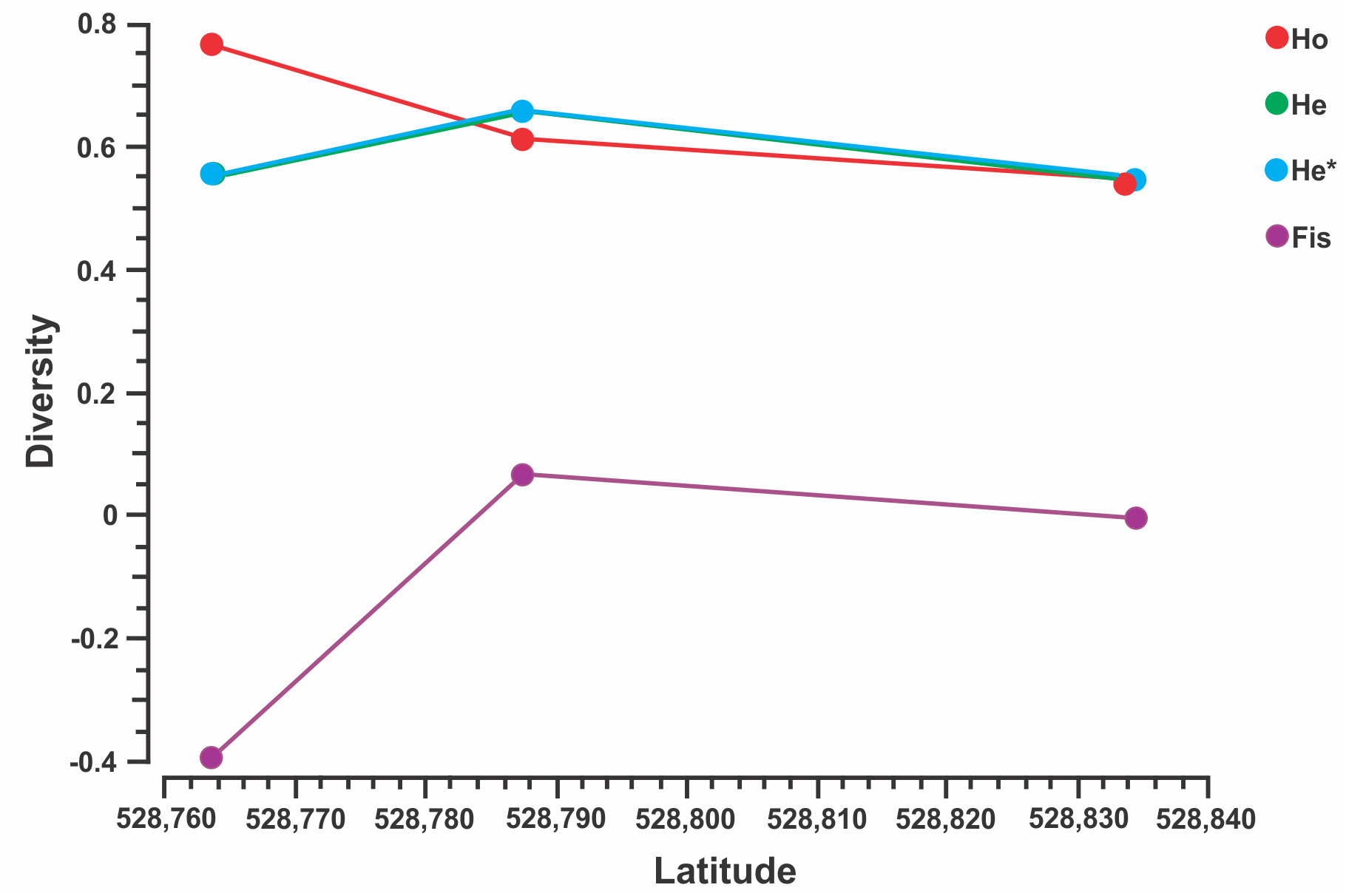

Supplement: Figure S5 — Diversity gradient plot showing observed heterozygosity (HO), expected heterozygosity (HE), expected heterozygosity corrected for small sample sizes (HE), and inbreeding (FIS). (TIF) [file pone.0103595.s005.tif]

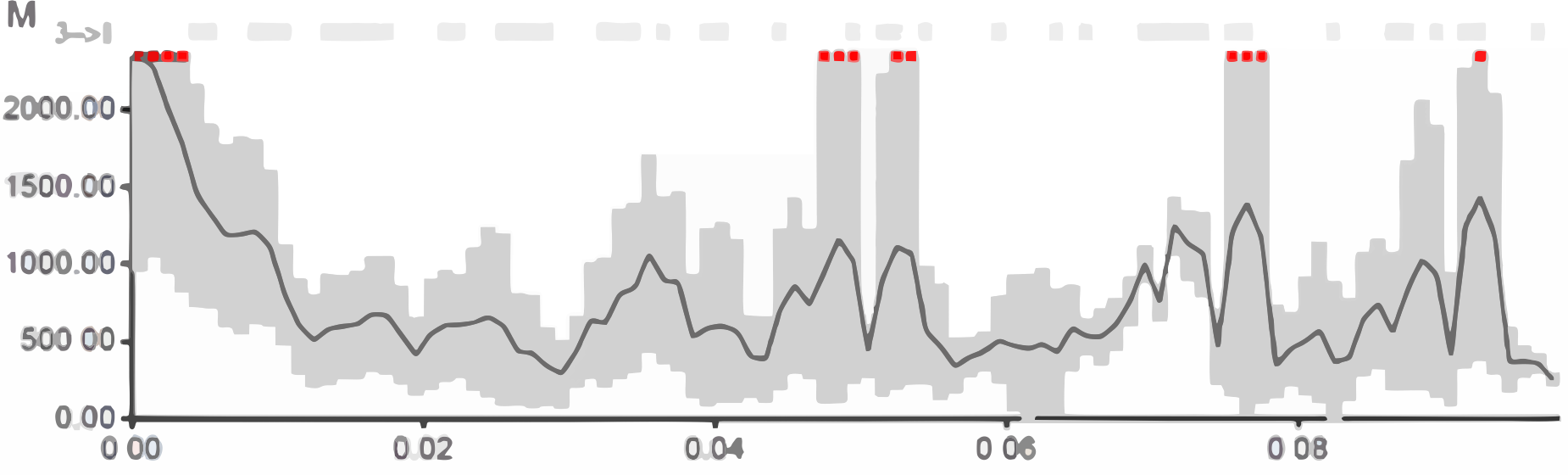

Supplement: Figure S6 — Skyline plot of a population that recently increased strongly, the time is in units of mutation scaled generations. (TIF) [file pone.0103595.s006.tif]
