# Supplementary material for: Microhabitat Types Promote the Genetic Structure of a Micro-Endemic and Critically Endangered Mole Salamander (Ambystoma leorae) of Central Mexico
Source: PLoS One. 2014 Jul 30;9(7):e103595. doi: 10.1371/journal.pone.0103595 (PMC4116214; doi:10.1371/journal.pone.0103595)
Supplement: Table S1 — Eigenvalues of each factor and the cumulative score of each factor. In bold are the most important values. (DOCX) [file pone.0103595.s007.docx]

|  | Eigenvalue | % Total | Cumulative | Cumulative |
| --- | --- | --- | --- | --- |
| 1 | **3.57914** | **39.76830** | **3.57914** | **39.76830** |
| 2 | **2.19411** | **24.37901** | **5.77325** | **64.14731** |
| 3 | **1.30767** | **14.52967** | **7.08092** | **78.67698** |
| 4 | 0.84888 | 9.43204 | 7.92981 | 88.10902 |
| 5 | 0.60380 | 6.70890 | 8.53361 | 94.81792 |
