# Supplementary material for: Microhabitat Types Promote the Genetic Structure of a Micro-Endemic and Critically Endangered Mole Salamander (Ambystoma leorae) of Central Mexico
Source: PLoS One. 2014 Jul 30;9(7):e103595. doi: 10.1371/journal.pone.0103595 (PMC4116214; doi:10.1371/journal.pone.0103595)
Supplement: Table S2 — Scores of each microhabitat characteristics of the factor analysis components. In bold are the most important variables. (DOCX) [file pone.0103595.s008.docx]

|  | Factor | Factor | Factor | Factor | Factor |
| --- | --- | --- | --- | --- | --- |
| COHAB1 (%) | 0.17190 | **0.93311** | 0.16491 | -0.02226 | 0.04640 |
| COHERB2 (%) | 0.07036 | **-0.96236** | -0.02214 | 0.01107 | -0.16723 |
| COVEGR (%) | **0.89730** | 0.22776 | 0.16118 | 0.28851 | -0.04021 |
| COSTONR (%) | **-0.88118** | -0.14118 | 0.15230 | 0.02397 | -0.37877 |
| Clear (%) | **-0.78969** | 0.21214 | -0.35752 | -0.35134 | 0.21077 |
| DEPR | -0.28688 | -0.62886 | -0.14107 | -0.60034 | -0.05654 |
| WIDER | 0.05901 | 0.13919 | 0.27649 | 0.02863 | 0.93364 |
| TEMPOUTR | -0.19141 | 0.04853 | 0.07350 | -0.94692 | -0.00809 |
| TEMPR | 0.13647 | 0.24136 | **0.84553** | -0.06132 | 0.42215 |
| Expl.Var | 2.38079 | 2.38911 | 1.02136 | 1.46953 | 1.27279 |
| Prp.Totl | 0.26453 | 0.26545 | 0.11348 | 0.16328 | 0.14142 |
