# Supplementary material for: Microhabitat Types Promote the Genetic Structure of a Micro-Endemic and Critically Endangered Mole Salamander (Ambystoma leorae) of Central Mexico
Source: PLoS One. 2014 Jul 30;9(7):e103595. doi: 10.1371/journal.pone.0103595 (PMC4116214; doi:10.1371/journal.pone.0103595)
Supplement: Table S3 — Table output of the Evanno method results. In bold is the largest value in the Delta K column. (DOCX) [file pone.0103595.s009.docx]

| **K** | **Reps** | **Mean LnP(K)** | **Stdev LnP(K)** | **Ln'(K)** | **\|Ln''(K)\|** | **Delta K** |
| --- | --- | --- | --- | --- | --- | --- |
| 1 | 10 | -2034.300 | 1.103 | — | — | — |
| 2 | 10 | -1986.560 | 2.351 | 47.740 | 13.780 | 5.859 |
| **3** | **10** | **-1925.040** | **5.481** | **61.520** | **155.470** | **28.360** |
| 4 | 10 | -2018.990 | 30.337 | -93.950 | 53.490 | 1.763 |
| 5 | 10 | -2059.450 | 40.340 | -40.460 | 91.030 | 2.256 |
| 6 | 10 | -2190.940 | 63.500 | -131.490 | 148.500 | 2.338 |
| 7 | 10 | -2173.930 | 96.683 | 17.010 | 125.200 | 1.294 |
| 8 | 10 | -2282.120 | 120.896 | -108.190 | 26.040 | 0.215 |
| 9 | 10 | -2416.350 | 76.454 | -134.230 | 169.990 | 2.223 |
| 10 | 10 | -2380.590 | 111.065 | 35.760 | — | — |
