# Supplementary material for: Microhabitat Types Promote the Genetic Structure of a Micro-Endemic and Critically Endangered Mole Salamander (Ambystoma leorae) of Central Mexico
Source: PLoS One. 2014 Jul 30;9(7):e103595. doi: 10.1371/journal.pone.0103595 (PMC4116214; doi:10.1371/journal.pone.0103595)
Supplement: Table S4 — Microenvironmental conditions range. (DOCX) [file pone.0103595.s010.docx]

| **Variable** | **Range** |
| --- | --- |
| COHAB1 | 50-100% |
| COHERB2 | 0-40% |
| COVEGR | 0-97% |
| COSTONR | 0-97.50% |
| DEPR | 0.35-0.47m |
| WIDER | 1.35-3.75m |
| TEMPOUTR | 10-12.5°C |
| TEMPR | 12-18.5°C |
